# Advanced Neuroimaging Role in Traumatic Brain Injury: A Narrative Review

**DOI:** 10.3389/fnins.2022.872609

**Published:** 2022-04-13

**Authors:** Ling Hu, Siyu Yang, Bo Jin, Chao Wang

**Affiliations:** ^1^Department of Ultrasound, Hangzhou Women’s Hospital, Hangzhou, China; ^2^Department of Radiology, The Second Affiliated Hospital, Zhejiang University School of Medicine, Hangzhou, China; ^3^Department of Neurology, Zhejiang Provincial People’s Hospital, People’s Hospital of Hangzhou Medical College, Hangzhou, China

**Keywords:** traumatic brain injury, neuroimaging, ultrasound, magnetic resonance imaging, molecular imaging

## Abstract

Traumatic brain injury (TBI) is a common source of morbidity and mortality among civilians and military personnel. Initial routine neuroimaging plays an essential role in rapidly assessing intracranial injury that may require intervention. However, in the context of TBI, limitations of routine neuroimaging include poor visualization of more subtle changes of brain parenchymal after injury, poor prognostic ability and inability to analyze cerebral perfusion, metabolite and mechanical properties. With the development of modern neuroimaging techniques, advanced neuroimaging techniques have greatly boosted the studies in the diagnosis, prognostication, and eventually impacting treatment of TBI. Advances in neuroimaging techniques have shown potential, including (1) Ultrasound (US) based techniques (contrast-enhanced US, intravascular US, and US elastography), (2) Magnetic resonance imaging (MRI) based techniques (diffusion tensor imaging, magnetic resonance spectroscopy, perfusion weighted imaging, magnetic resonance elastography and functional MRI), and (3) molecular imaging based techniques (positron emission tomography and single photon emission computed tomography). Therefore, in this review, we aim to summarize the role of these advanced neuroimaging techniques in the evaluation and management of TBI. This review is the first to combine the role of the US, MRI and molecular imaging based techniques in TBI. Advanced neuroimaging techniques have great potential; still, there is much to improve. With more clinical validation and larger studies, these techniques will be likely applied for routine clinical use from the initial research.

## Background

Traumatic brain injury (TBI) is a common source of morbidity and mortality among civilians and military personnel. It is estimated that the global incidence of TBI is at 10 million cases annually (Hyder et al., 2007). A particularly high incidence of TBI is seen among military personnel. Overall TBI admission rates ranged from 24.6 to 41.8% per 10,000 soldier-years in the Afghanistan and Iraqi wars (Wojcik et al., 2010).

For modern service members, chronic exposure to repetitive shots from advanced weapon systems near the head is a common cause of sub-concussive forces. Acute injury, such as explosive injury, has a lower incidence but varies more in TBI severity range from mild to severe (Table 1). Glasgow Coma Scale (GCS) is one of the more common methods of evaluating TBI severity. Of the three subtypes of TBI, mild TBI is the most common subtype, accounting for approximately 75–90% (Fehily and Fitzgerald, 2017). However, mild TBI is often the most challenging subtype to diagnose, in part because of a lack of clinically objective and measurable signs of brain injury. Long-term sequelae of TBI ranges from mild cognitive impairment to severe disability. In many cases, the diagnosis becomes clear when a patient presents with physical manifestations of intracranial injury and routine neuroimaging findings, indicating that the injury may require urgent surgical intervention and medication, such as intra-axial and extra-axial hematoma resulting in life-threatening cerebral herniation. Routine neuroimaging shortly after brain injury may also provide information on future prognosis, such as the early mortality and late morbidity (Maas et al., 2005). Routine neuroimaging techniques for the clinical evaluation of TBI include head computed tomography (CT), ultrasound (US) (He et al., 2013), and conventional magnetic resonance imaging (MRI) sequences (Shetty et al., 2016) (e.g., T1-weighted imaging (T1WI), T2-weighted imaging (T2WI), fluid attenuated inversion recovery (FLAIR), susceptibility weighted imaging (SWI), diffusion-weighted imaging (DWI)), which can assist with detecting acute intracranial injuries (e.g., hematoma) and chronic effects of TBI (e.g., encephalomalacia, hemosiderin). However, in the context of TBI, limitations of routine neuroimaging include poor visualization of more subtle changes of brain parenchymal after injury, poor prognostic ability, and inability to analyze cerebral perfusion, metabolite and mechanical properties.

**TABLE 1:** Guidelines for the diagnosis of TBI severity.

	Mild TBI	Moderate TBI	Severe TBI
GCS	13–15	9–12	3–8
LOC	≤30 min	>30 min but <24 h	≥24 h
PTA	≤24 h	>24 h but <7 d	≥7 d
Imaging findings	No CT abnormalities	Abnormal CT findings	Abnormal CT findings

*GCS, Glasgow coma scale; LOC, Loss of consciousness; PTA, Posttraumatic amnesia; TBI, Traumatic brain injury; min, minutes; h, hours; d, days; CT, Computed tomography.*

With the development of modern neuroimaging techniques, it is possible to detect and display more subtle brain injury changes. Routine neuroimaging findings might be negative for mild TBI. However, advanced neuroimaging techniques are perhaps even more promising in diagnosis, prognostication, and eventually impacting treatment. Advances in neuroimaging techniques have shown potential, including (1) US based techniques (contrast-enhanced US, intravascular US, and US elastography), (2) MRI based techniques (diffusion tensor imaging (DTI), magnetic resonance spectroscopy (MRS), perfusion weighted imaging (PWI), and magnetic resonance elastography (MRE), functional MRI), and (3) molecular imaging based techniques (positron emission tomography (PET) and single photon emission computed tomography (SPECT)). Therefore, in this review, we aim to summarize the role of advanced neuroimaging techniques in the evaluation and management of TBI.

## Search Strategy and Selection Criteria

This is a narrative review where search parameters were used. Searches of PubMed were performed to obtain the data and articles for this review. The search terms used were “traumatic brain injury,” “TBI,” “magnetic resonance imaging,” “ultrasound,” “ultrasound elastography,” “USE,” “MRI,” “functional MRI,” “fMRI,” “diffusion tensor imaging,” “DTI,” “magnetic resonance spectroscopy,” “MRS,” “perfusion weighted imaging,” “PWI,” “magnetic resonance elastography,” “MRE,” “positron emission tomography,” “PET,” “single photon emission computed tomography,” and “SPECT.” Abstracts and articles were reviewed and included only if they met our criteria of discussing the role of neuroimaging techniques in evaluating TBI. There was no date limitation to the articles included.

## Advanced Ultrasound Based Techniques

### Contrast-Enhanced Ultrasound

Contrast-enhanced US is particularly useful in assessing microvascular perfusion (Bailey et al., 2017; Hwang et al., 2017). Compared with conventional US, contrast-enhanced US requires intravenous contrast agent (microbubble) to enhance visualization of anatomical details, which can offer high soft tissue contrast and improve diagnostic sensitivity. Using contrast-enhanced US, the quantifiable perfusion kinetic parameters can be obtained from the wash-in curve, wash-out curve, and destruction-replenishment curve after intravascular microbubble injection, which can be used as a valuable clinical tool for monitoring vasospasm and reperfusion after TBI. This technique can be performed at the bedside quickly and provide serial monitoring of brain perfusion abnormalities, particularly for hemodynamically unstable patients.

Compared with conventional US, intraoperative contrast-enhanced US during open craniectomy shows more clearly defined border and significantly larger measured size of the trauma lesion in TBI patients (He et al., 2013). Contrast-enhanced US provides more detailed information about vascular perfusion, which help neurosurgeon distinguish contused brain tissue (with low or absent enhancement) from healthy brain tissue (with homogeneous enhancement). In 66% (21 of 32) of the cases in a study, the procedure enlarged the area originally planned to be removed. In the patients with TBI, the severity of the trauma lesion was reclassified and surgical intervention was redesigned with this technique in 21 (21/32, 66%) cases (He et al., 2013), indicating that contrast-enhanced US helps neurosurgeons effectively remove hematoma, preserve normal brain tissue, and prevent vascular injury during surgery. Furthermore, a study by Heppner et al. has demonstrated contrast-enhanced US has the potential for both intraoperative and bedside evaluation of cerebral perfusion in patients with TBI, which may be suitable for assessing its responses to decompressive craniotomy and therapies aimed at preventing secondary ischemia (Heppner et al., 2006). Through the temporal bone window, contrast-enhanced US also plays a promising role in perfusion assessment even without craniotomy. Using contrast-enhanced US, Eyding et al. demonstrated the difference between the core region of no perfusion and the surrounding region of hypoperfusion could be detected through the temporal bone window (Eyding et al., 2004). In addition, Eyding et al. evaluated the perfusion of cerebral parenchyma, they found the hypoperfusion of cerebral parenchyma was associated with the functional status in the patients of acute stroke (Eyding et al., 2006). Contrast-enhanced US can provide important information on the pathological changes of vasogenic edema and vascular perfusion after brain injury. With no concerns for adverse reactions in the brain, contrast-enhanced US has been approved by the Food and Drug Administration in America. Nevertheless, more future preclinical and clinical studies are needed to finally generalize this technique for the patients with TBI.

### Intravascular Ultrasound

Intravascular US is a rapidly evolving new imaging technique that can be applied for a multitude of both diagnostic and interventional purposes (Marteslo et al., 2020). Compared to conventional US, intravascular US enables real-time cross-sectional imaging of the vasculature from inside the vessel to the adjacent extravascular structures without the problem of motion artifacts or volume averaging, which may play a promising role in endovascular treatment of vascular injuries in TBI.

In patients with equivocal CT angiography (CTA) in suspected blunt traumatic aortic injury, intravascular US has been revealed to be more sensitive than CTA (Azizzadeh et al., 2011). Stager et al. reported successful endovascular treatment of traumatic internal carotid artery pseudoaneurysm with bare metal stenting by intravascular US guidance (Stager et al., 2011). The neck of the pseudoaneurysm spanned 2 cm by intravascular US compared with 1 cm by angiography. Therefore, based on intravascular US findings, a longer stent was used, and 1-year follow-up CTA showed no residual pseudoaneurysm. As shown in the study of Stager et al., intravascular US enables clinicians to more accurately visualize anatomic details of traumatic vascular injuries, thus contributing to making an optimal treatment decision. Similarly, a patient with traumatic carotid-cavernous fistula was successfully monitored by intravascular US and accurate coil embolization was achieved (Nishio et al., 2009). Although intravascular US has shown the high diagnostic accuracy in large vessels, further application of intravascular US in TBI would require a smaller US transducer that can be placed in the cerebral vessels (Peng et al., 2021). With the development of hardware size, portability and resolution, intravascular US will play a critical role in traumatic vessel imaging following TBI.

### Ultrasound Elastography

Ultrasound elastography is an imaging technique sensitive to tissue stiffness. In recent years, this technique has been further developed and refined to achieve quantitative evaluation of tissue stiffness. US shear-wave elastography generates a map of the Young’s modulus, a property describing the stiffness of the tissue of interest (Sigrist et al., 2017). US elastography takes advantage the altered elasticity of in soft tissue that occur as a result of specific pathological or physiological processes (Shiina et al., 2015). Since the mechanical properties of living tissue alter under different pathological or physiological conditions, US elastography can detect these alterations and has been clinically applied in the liver, breast, thyroid, kidney, prostate diseases (Sigrist et al., 2017).

Using US elastography, Xu et al. found a decreased stiffness values (as measured by Young’s modulus) due to subsequent tissue edema in the ipsilateral hemisphere of cerebral ischemic infarction (Xu et al., 2013). Furthermore, Xu et al. applied US elastography at the craniectomy site after injury and compared the hemispheric stiffness values in a rodent model of mild TBI (Xu et al., 2014). They found that there was an ipsilateral decrease and contralateral increase of the stiffness values by 24 h after injury. The observed decrease in the stiffness values in the injured hemisphere is consistent with the findings from MRE studies on TBI models of mice that showed a significant reduction in the stiffness of the injured region immediately after injury (Boulet et al., 2011, 2013). Tissue stiffness values are sensitive to the tissue’s net fluid content. For example, all else being equal, the greater the percentage of fluid in the tissue, the lower the measured stiffness, and the less fluid, the greater stiffness (Tanter et al., 2008). Therefore, the decreased stiffness values in the injured hemisphere were mainly attributed to the edema formation ipsilateral to injury by 24 h (Xu et al., 2014). Moreover, Xu et al. explained that the increased stiffness values in the contralateral hemisphere were attributed to reduced cerebral perfusion pressure and cortical perfusion contralateral to the injury. Reduced blood flow to the brain parenchyma resulted in a reduction in the fluid content and an increased stiffness values of the brain parenchyma. In addition, transcranial application of a form of US called vibro-acoustography (VA) was applied to detect cerebral changes in rat following TBI (Suarez et al., 2015). The VA technique uses ≥2 US vibrations at slightly different frequencies to characterize the material properties of the object based on its acoustic response to the applied vibration. From that study, the preliminary findings revealed that TBI model rats showed decreased acoustic emissions relative to sham-TBI rats. In the future, more studies are needed to understand the exact mechanism behind the changes in the tissue stiffness values over a chronic time course. Additionally, future studies of clinical applications are needed to the human brain *in vivo* and further improvements in US resolution may make the US elastography useful in real clinical application.

## Advanced Magnetic Resonance Imaging Based Techniques

### Diffusion Tensor Imaging

Diffusion tensor imaging, a more advanced MRI technique based on DWI, measures the diffusion of water in multiple spatial directions and provides information about axon bundles (Mukherjee et al., 2008). DTI can calculate diffusion with a variety of parameters. Of these parameters, the commonly reported parameters are fractional anisotropy (FA), which quantifies the asymmetry of water diffusion, and mean diffusivity/apparent diffusion coefficient (MD/ADC), which measures the average magnitude of water diffusion.

Using DTI, Arfanakis et al. showed the white matter damage could be detected within 24 h following TBI (Arfanakis et al., 2002). Thus, they suggested DTI may be a promising technique for detecting diffuse axonal injury *in vivo*. Since then, more and more DTI studies of TBI have appeared. The reported results vary widely depending on the TBI severity and imaging time after injury. In the acute mild TBI, several DTI studies showed increased FA of corpus callosum, which may be caused by cytotoxic edema following TBI (Bazarian et al., 2007; Wilde et al., 2008). To explore the pattern of the impact of moderate to severe TBI on microstructural development, Wilde et al. demonstrated microstructural FA decrease and ADC increase over 18 months after injury (Wilde et al., 2012). A meta-analysis of 44 studies found that moderate to severe TBI resulted in larger white matter microstructural damage than mild TBI (Wallace et al., 2018a). However, the findings from DTI performed in the acute, subacute and chronic intervals did not show any time-dependent changes (Wallace et al., 2018a).

In addition, using DTI, a number of studies focused on the association between the cognitive and functional outcomes of mild to severe TBI and white matter microstructural changes. In patients with very mild TBI, to determine the association between frontal white matter damage and acute executive function impairment, a study by Lipton et al. showed that decreased FA in the dorsolateral prefrontal cortex (DLPFC) was significantly correlated with worse executive function performance (Lipton et al., 2009). A meta-analysis investigated the relationship between DTI and cognitive outcomes in TBI patients (Wallace et al., 2018b). That meta-analysis included a total of 20 DTI studies and reported that in most brain regions, a high FA and low MD/ADC were found to be associated with better cognitive performance, particularly memory and attention (Wallace et al., 2018b). Furthermore, in mild TBI patients, Yuh et al. utilized the DTI findings to predict 3- and 6-month Glasgow outcome scale-extended (GOS) outcomes. They demonstrated significantly reduced FA was correlated with worse 3- and 6-month GOS outcomes (Yuh et al., 2014). After mild TBI, a part of patients experienced persistent post-concussion symptoms (PPCS) for several months to years (Polinder et al., 2018). Compared to the patients who recovered and healthy controls, mild TBI patients who developed PPCS showed more severely damaged white matter microstructural integrity after brain injury (Stenberg et al., 2021). In severe TBI, high FA at the initial scan could predict favorable GOS outcome of 1 year, and increased FA in the internal capsule and in centrum semiovale during follow-up of 1 year, particularly in patients with favorable GOS outcome (Sidaros et al., 2008). The increased FA over time was hypothesized to be secondary to axonal recovery or axonal regrowth during later recovery (Sidaros et al., 2008). Although the time interval following TBI, brain areas examined, magnetic strength and analysis methods were various in these previous studies, most studies have shown that DTI is a sensitive neuroimaging technique detecting white matter microstructural damage in TBI. Consensus guidelines for the application of DTI parameters are needed in future TBI studies.

### Magnetic Resonance Spectroscopy

Magnetic resonance spectroscopy relies on detecting magnetic field interactions between protons according to their Larmor resonance frequencies (Wintermark et al., 2015). MRS can quantify chemicals in a certain tissue and manage to generate a spectrum of the signal intensities of different metabolites (Astrakas and Argyropoulou, 2016). The metabolites include N-acetylaspartate (NAA) for neuronal viability, creatine (Cr) for cellular energy utilization, choline (Cho) for membrane turnover, lactate (Lac) for anaerobic metabolism, glutamate/glutamine (Glx) for excitatory neurotransmission, and myoinositol (mI) for membrane turnover and reactive gliosis (Garnett et al., 2000).

Quantifying the metabolites, MRS is thought to be more sensitive to detect and manage TBI, particularly when they occur in the absence of visible injury on routine anatomic imaging. In both acute and chronic phases, NAA/Cr ratios are decreased in mild TBI patients (Henry et al., 2011). Further, a longitudinal study showed NAA levels was decreased and remained low in patients with unfavorable outcome, while NAA levels recovered in patients with favorable outcome (Signoretti et al., 2002). A meta-analysis by Gardner et al. identified 11 studies of mild TBI and found that there were significant differences in MRS after injury in most studies (Gardner et al., 2014). They concluded that metabolic disruption remains even after the resolution of symptoms. In addition, in mild TBI patients, a prospective longitudinal study by George et al. revealed a positive association between Cr levels in the centrum semiovale and chronic neurocognitive performance, as measured half year after injury (George et al., 2014). For moderate to severe TBI, early decline in NAA levels in subcortical brain regions was an early indicator of tissue injury, which could further predict long-term neurocognitive outcomes (Holshouser et al., 2019). Similarly, reduced NAA/Cho ratios and NAA/Cr ratios in the acute period were correlated with poor neurologic outcome half year or more after injury (Aaen et al., 2010). In addition to NAA, Cho and Cr, mI was found to be elevated following TBI and associated with a poor neurologic outcome (Ashwal et al., 2004). The mI elevation was attributed to proliferative astrogliosis or disturbance in osmotic function.

Taken together, decreases in the NAA and Cr, as well as increases in Cho, usually occur in TBI patients, and the magnitude of these metabolic changes correlate with the severity of TBI and also with functional outcomes. MRS has shown its potential role in detecting the metabolic changes of TBI. However, clinical MRS applications for TBI are limited because of their significant overlap with the metabolites changes that are also seen in many other brain disorders, which reduces the specificity of MRS for TBI. Therefore, more future studies are needed to determine how to best apply this technique.

### Perfusion Weighted Imaging

Traumatic brain injury is related to impaired cerebral vascular auto-regulation function, vascular injury and increased blood-brain barrier permeability, then resulting in the changes of cerebral blood flow (CBF), ischemia and even infarction (Bigler and Maxwell, 2012). For these mechanisms, the studies using perfusion imaging have long been a target technique for TBI. Many imaging tools have been used in the previous studies, such as CT perfusion (CTP) (Wintermark et al., 2004), xenon-enhanced CT (Xe-CT) (Rostami et al., 2014), SPECT (Abu-Judeh et al., 1998; Davalos and Bennett, 2002), PET (Yamaki et al., 1996), and dynamic susceptibility contrast (DSC) PWI (Garnett et al., 2001), and arterial spin-labeling (ASL) PWI (Ge et al., 2009; Grossman et al., 2013). However, perfusion imaging techniques using CT, SPECT, and PET all have radiation. For example, previous study has demonstrated that single head CT scan generates about 2 mSv of radiation, whereas a CT radiation dose of 10 mSv is associated with fatal cancer in 1 per 2,000 patients (Costello et al., 2013). Multiple scans may increase the risk of malignancy. However, MRI can be performed without any radiation. Moreover, MRI has higher soft tissue resolution and can may provide a higher level of anatomic detail of brain injury than CT. DSC-PWI requires injection of intravenous contrast agent, while ASL-PWI does not, and is based on labeling endogenous water in the blood and using it as a tracer (Douglas et al., 2018). Using DSC-PWI, Garnett et al. measured CBV in both contused and normal-appearing brain tissue. Notably, despite the small sample size, reduced CBV was not only detected in contused regions but also normal-appearing brain tissue on DSC-PWI. Furthermore, the reduced CBV correlated with worse clinical outcomes (Garnett et al., 2001). Using DSC-PWI, in mild TBI patients, Papadaki et al. found significantly reduced CBF in DLPFC, putamen, and hippocampus, and reduced CBF correlated with psychoemotional outcomes (e.g., anxiety and depression) (Papadaki et al., 2021). ASL-MRI is an alternative MRI perfusion technique. Using ASL-PWI, in mild TBI patients, CBF was significantly reduced in the thalamus relative to healthy controls (Ge et al., 2009; Grossman et al., 2013). And the reduction of thalamic CBF was strongly correlated with neurocognitive impairment (Ge et al., 2009). In addition, using ASL-PWI, Li et al. investigated not only region CBF but also CBF connectivity features in acute mild TBI patients, they reported both regional CBF abnormalities and CBF connectivity deficits in these patients (Li et al., 2020). Although the dynamics of these studies are too weak to adequately assess the prognostic power of ASL perfusion in mild TBI patients, the results are interesting. These studies initially demonstrate the potential role of PWI in TBI evaluation. In the future, more prospective and longitudinal studies are needed to determine the role of PWI in evaluating TBI.

### Magnetic Resonance Elastography

Magnetic resonance elastography is scanned with an MRI pulse sequence that generates propagating sound waves and a measurable tissue displacement (Muthupillai and Ehman, 1996). The pulses needed for MRE are created in an experimental model whose driver converts compressed air into motion and transmits it to a pillow beneath the patient’s head (Klatt et al., 2015). Then, the displacement information is converted into an elastic graph, and the intrinsic cerebral viscoelasticity of the brain tissue being tested. Using MRE, the mechanical property of the brain tissue can be visualized and measured non-invasively. MRE is considered a particularly sensitive imaging tool that may increase the potential for early diagnosis of neurological disorders (Hiscox et al., 2016), which has been applied to a number of neurological disorders, such as multiple sclerosis (Wuerfel et al., 2010), Alzheimer’s disease (Murphy et al., 2016), frontotemporal dementia (Huston et al., 2016), Parkinson’s disease (Lipp et al., 2013), amyotrophic lateral sclerosis (Romano et al., 2014). With MRE, several studies of preclinical models have indicated its potential usefulness in TBI. The mechanical properties of the brain (as measured by MRE) have been found to change following brain injury in living rat and mice (Alfasi et al., 2013; Boulet et al., 2013). Furthermore, using a TBI mouse model, a longitudinal study of MRE demonstrated that the elastic modulus of the injured brain tissue was higher than that of the contralateral hemisphere 1 h after injury (Feng et al., 2017). However, the elastic modulus decreased 1 day after injury, and recovered to be close to the brain tissue in the contralateral hemisphere in 7 days. Although more preclinical and clinical validations are needed for this emerging modality in the future, MRE technique holds the promise in measuring the mechanical properties of the brain *in vivo* through non-invasive scans, potentially improving prognostication.

### Functional Magnetic Resonance Imaging

Functional MRI relies on blood oxygen level-dependent (BOLD) imaging, which reflects hemodynamic response associated with neural activity. Transient local increases in neural activity lead to changes in the ratio of oxygenated-to-deoxygenated hemoglobin, which, in turn, affects the MRI signal response (Logothetis et al., 2001). Functional MRI has been widely applied to investigate the spontaneous activity of the human brain *in vivo*. It has been well-established that human consciousness involves interconnected circuits, of those, one most important network is called the default mode network (DMN) (Mason et al., 2007). Using resting-state functional MRI, Nathan et al. revealed the DMN dysfunction in the military mild TBI patients (Nathan et al., 2017). Furthermore, Zhou et al. investigated the integrity of the DMN, and found the decreased posterior DMN connectivity correlated positively with neurocognitive dysfunction, while the increased anterior DMN connectivity correlated negatively with posttraumatic symptoms (Zhou et al., 2012). Cognitive fatigue is one of the most reported symptoms following TBI (Beaulieu-Bonneau and Ouellet, 2017). Recently, Bruijel et al. found that cognitive fatigue was linked to DMN connectivity and was differently associated with striatal connectivity in moderate-severe TBI patients relative to healthy controls (Bruijel et al., 2022). This highlights the dynamic nature of DMN and suggests how changes in one brain area may trigger compensatory neural responses in other brain areas to achieve homeostasis of neural information exchange (Andrews-Hanna et al., 2010).

In addition, DLPFC plays a critical role in cognitive control (MacDonald et al., 2000). Using task-based functional MRI, Witt et al. demonstrated that mild TBI patients exhibited reduced BOLD activity in the DLPFC (Witt et al., 2010). DLPFC also serves as a part of the pain modulatory system. Using pain anticipation task, Strigo et al. demonstrated that mild TBI patients required greater DLPFC and subcortical engagement and greater use of modulatory resources than healthy controls to achieve comparable control over aversive experiences during pain (Strigo et al., 2014). Cerebral functioning and behavioral performance are influenced by pain following TBI. Gosselin et al. found that mild TBI patients with severe pain showed decreased BOLD activation of the DLPFC during the working memory task and poorer task performance, while patients with other injuries and severe pain showed that pain was associated with increased BOLD activation of the DLPFC and not correlated with task performance (Gosselin et al., 2012). The authors suggest that the interactions among pain, cognition, and cerebral functioning could not easily be generalized from one type of injury to another type. However, it should be noted that the possibility of partial overlap between these functional networks cannot be ignored in TBI patients. Functional MRI is a very promising technique for evaluating the cerebral functioning change in TBI, but further rigorous testing is needed before these results can be generalized clinically.

## Advanced Molecular Imaging Based Techniques

### Positron Emission Tomography and Single Photon Emission Computed Tomography

Positron emission tomography and SPECT are performed with tracer molecules tagged with radioisotopes. Early researches employing PET and SPECT in TBI mainly focused on perfusion imaging. TBI is related to impaired cerebral vascular autoregulation and raised blood-brain barrier permeability, resulting in CBF changes, ischemia and even infarction (Bigler and Maxwell, 2012). In patients with mild and moderate TBI, early SPECT studies showed hypoperfusion (Jacobs et al., 1994; Abu-Judeh et al., 1998; Hofman et al., 2001; Davalos and Bennett, 2002). SPECT studies only allow qualitative comparisons between brain regions, whereas PET is quantitatively accurate. Similarly, hypoperfusion was also detect after severe TBI in early PET study (Yamaki et al., 1996). In addition, with metabolic imaging, primarily with fluorodeoxyglucose (FDG)-PET, previous studies showed a broad pattern of prolonged brain hypometabolism that may last for days to months following mild TBI (Byrnes et al., 2014).

Although currently these PET and SPECT techniques have limited clinical application in the patients with TBI, these techniques have a lot of potential. In recent years, PET tracers have the potential to target amyloid plaques and tau proteins (Small et al., 2013; Hong et al., 2014). Chronic traumatic encephalopathy (CTE) is a neurodegenerative disease linked to chronic TBI. With amyloid PET, a study of small sample has demonstrated an increased Aβ deposition in the cortical gray matter and the striatum, but not in the thalamus or white matter in mild-to-severe TBI up to 1 year after injury (Hong et al., 2014). With tau PET, the patients with CTE have been found to show increased tau levels compared to healthy controls (Stern et al., 2019). These previous studies recruited only a small sample of subjects and require further validation in the future studies before routine clinical application.

## Conclusion

Advanced neuroimaging techniques (i.e., MRI-based, US-based and molecular imaging-based techniques) have great potential; still, there is much to improve. With more clinical validation and larger studies, these techniques will be likely applied for routine clinical use from the initial research. Several specific injuries of TBI hold promise for future innovation. Among them, mild TBI have attracted much attention due to their high incidence and lack of detection on routine imaging. The ability to predict functional prognosis using advanced neuroimaging techniques is another promising area of active research. With innovation and improvements of technology, advanced neuroimaging techniques will be further developed to likely guide which patients will benefit from some specific therapy and also help track their response to therapy.

For US-based advanced techniques (i.e., contrast-enhanced US, intravascular US, and US elastography), although imaging scientists and clinicians were unable to use US imaging as a first-line and routine assessment tool for TBI patients due to a lack of an adequate acoustic imaging window in the past, there have been a lot of new advances in US-based imaging as described above. US-based imaging can play a complementary role in evaluating TBI in addition to routine imaging. Compared to MRI-based and molecular imaging-based techniques, US has several obvious inherent advantages, such as cost-effectiveness, portability, rapid availability, and lack of radiation. Therefore, in the future, with innovation of US equipment and further improvements of US resolution, US-based advanced techniques will play a critical role in the evaluation and management of TBI patients. For MRI-based advanced techniques (i.e., DTI, MRS, PWI, MRE, and functional MRI), multiple-modality and various analytical methods are available for analysis of the MRI-based data for population-based research. Nevertheless, there is a lack of effective analytical methods that can be clinically used to quantitatively analyze advanced MRI-based data in an individual subject or patient. In recent years, machine learning has been developed to make individual distinctions in a number of neuroimaging studies in many neurological or psychiatric disorders. Therefore, in the future, a novel approach of quantitative analysis with machine learning in the level of a single individual is needed before bringing these techniques into routine diagnostic use in standard clinical care. For molecular imaging-based techniques (i.e., PET and SPECT), previous studies were case reports or small sample studies, thus the findings require further validation in larger studies before routine clinical application. In the future, combining multimodal neuroimaging modalities, and developing multi-modal neuroimaging biomarkers in TBI will go a long way to providing more accurate diagnosis, stratification of disease severity of TBI as well as providing important indicators of treatment response and outcome.

## Author Contributions

LH carried out literature searches, did data interpretation, and writing. SY and BJ did literature searches and data interpretation. CW conceptualized the manuscript and did writing and editing. All authors read and approved the final manuscript.

## Conflict of Interest

The authors declare that the research was conducted in the absence of any commercial or financial relationships that could be construed as a potential conflict of interest.

## Publisher’s Note

All claims expressed in this article are solely those of the authors and do not necessarily represent those of their affiliated organizations, or those of the publisher, the editors and the reviewers. Any product that may be evaluated in this article, or claim that may be made by its manufacturer, is not guaranteed or endorsed by the publisher.
